# Small intrusions may help maintain Kīlauea’s lava lake

**DOI:** 10.1007/s00445-025-01847-8

**Published:** 2025-07-07

**Authors:** Taha Sadeghi Chorsi, Elisabeth Gallant, Lichen Forster, Jacqueline E. Dixon, Timothy H. Dixon

**Affiliations:** 1 School of Geosciences, University of South Florida, 4202 E. Fowler Avenue NES 107, Tampa, 33620-5250 FL USA; 2 Department of Geology, University of Hawai‘i at Hilo, 200 W. Kāwili, Hilo, 96720-4091 HI USA; 3 College of Marine Science, University of South Florida, 140 Seventh Ave. South, St. Petersburg, 33701 FL USA

**Keywords:** Kīlauea, Lava lake, Radar, Surface deformation

## Abstract

We collected rapid-repeat radar data capturing the deformation of the active Halema‘uma‘u summit lava lake surface on January 19, 2023, an otherwise quiescent period during the January–March 2023 eruption. Radar interferograms were generated every 90 s over a 90-min period of intermittent inflation using a scanning real aperture radar operating at Ku-band (17.4 mm wavelength). This technique allows observation of phenomena at a temporal scale and spatial resolution not previously possible. We model the intrusion as a shallow sill, 10 to 100 m below the lava lake surface. We suggest that frequent intrusions of such small volume pulses of gas-rich magma help to provide the flux of heat and mass necessary to compensate for cooling, outgassing, and recycling of dense degassed magma to deeper levels, sustaining the lava lake during periods of near-steady-state.

**Supplementary Information:**

The online version contains supplementary material available at 10.1007/s00445-025-01847-8.

## Introduction

Persistent lava lakes are rare; only a few volcanoes have hosted them in recent decades, including Mount Erebus, Antarctica (Dibble et al. 1984), Erta Ale, Ethiopia (Carniel et al. 2002), Masaya Volcano, Nicaragua (Rymer et al. 1998), Mt. Michael Volcano, South Sandwich Islands (Gray et al. 2019), Mount Nyiragongo, Democratic Republic of the Congo (Tazieff 1984, 1985), and Kīlauea Volcano, Hawai‘i (Barker et al. 2003; Swanson et al. 1979; Patrick et al. 2019). These lakes represent a dynamic balance between surface cooling and outgassing versus deeper recharge by hot, gas-rich magma (e.g., Lev et al. 2019; Patrick et al. 2019; Poland and Carbone 2016). Outgassing and cooling results in dense degassed magma that may drain back into summit magma reservoirs (e.g., Dixon et al. 1991; Suckale et al. 2018; Wallace and Anderson Jr 1998). Lava lakes can drain and refill, or even disappear completely, depending on the balance of these various processes. In the absence of fresh magma, lava lakes would likely cool and solidify within a few years to decades (Wright and Okamura 1977).

**Fig. 1:**
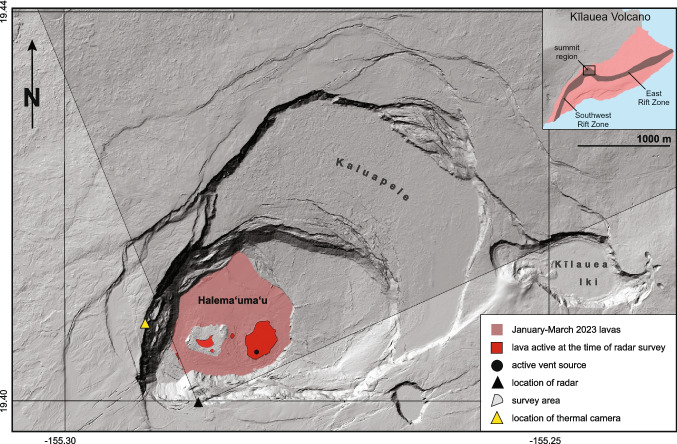
Map depicting the Kīlauea summit eruption status as of January 18, 2023, the day before our GPRI data collection. The eruptive vent inside Halema‘uma‘u crater lies within the active lava lake (red) on the eastern side of the crater floor. The radar location (black triangle) and its zoomed-in field of view are also shown. Thermal camera (F1cam) location is shown in yellow triangle (see Fig. 6)

The study of lava lakes not only provides insight into mechanisms controlling lava lake behavior but also helps answer broader questions related to the magmatic process (e.g., Anderson and Poland 2016; Lev et al. 2019; Patrick et al. 2019). For example, meters to tens of meters of motion of the lake surface over time scales of hours to years have been attributed to the interplay of two main processes: (1) changes in deeper magma reservoir pressure on timescales of days to years (e.g., change in magma supply rate); and (2) changes in shallow outgassing on timescales of minutes to hours (e.g., gas pistoning; Patrick et al. 2019). Degassing studies, in particular, have proven to be an especially fertile source of insight into the role of volatiles as they relate to eruptive styles and other magmatic processes (e.g., Crozier and Karlstrom 2022; Edmonds et al. 2013).

In this study, we use surface deformation data from a Gamma Portable Radar Interferometer (GPRI) to capture changes of the lava lake surface at Halema‘uma‘u summit crater, Kīlauea Volcano. The radar measurements were originally collected to generate a high-resolution digital elevation model (DEM) of the crater. However, during a 90-min observation period on January 19, 2023, we unexpectedly recorded a small surface deformation event. This event exhibited a maximum line-of-sight displacement of approximately $\sim$3 cm—much smaller than the gas pistoning events previously described in the literature. To our knowledge, this is the first study of lava lake deformation using high spatial and temporal resolution radar interferometry. Our new measurements provide insight into magma supply and outgassing processes at temporal and spatial scales not previously possible.

**Fig. 2:**
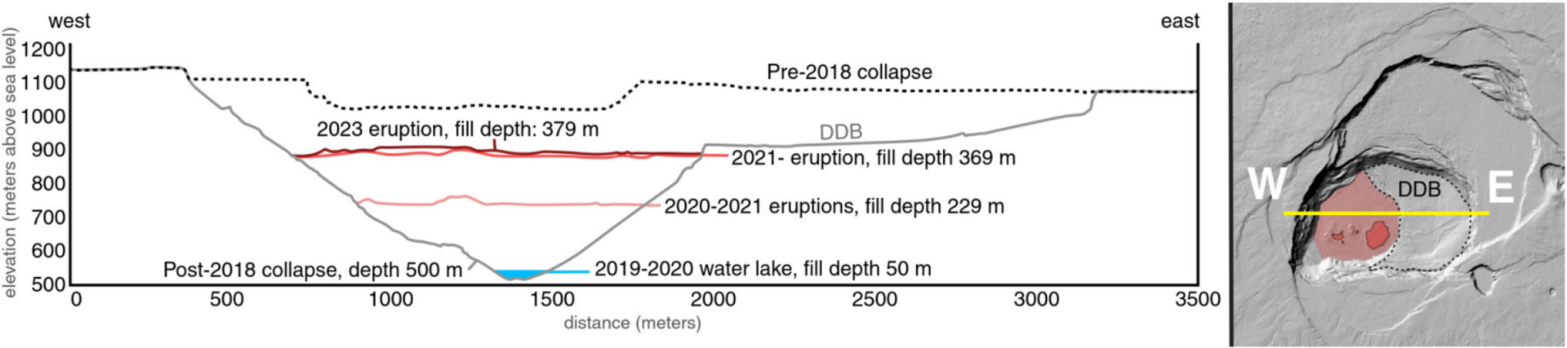
Caldera profiles for the periods before the 2018 caldera collapse (black dotted line), shortly after the 2018 collapse (gray), the December 2020–May 2021 eruption (pink), the September 2021–December 2022 eruption (red), and the 2023 eruption (brown) as modeled from the HVO overflight on January 17, 2023. The maximum depth of the 2019–20 Halema‘uma‘u water lake is shown in blue (USGS 2023a)

## Geologic setting

The present-day summit caldera of Kīlauea, Kaluapele, (Fig. 1) formed by collapse sometime between 1470 and 1510 A.D., following the large volume, effusive Kualoloa eruption (Swanson et al. 2012). Halema‘uma‘u, a pit crater within the Kaluapele summit caldera, has hosted many of the historical summit lava lakes described in both Mo‘olelo and written accounts (e.g., Desha 2000; Douglas 1834; Ellis 1827; Patrick et al. 2020).

In March 2008, an earthquake caused a collapse on the south wall and saw a return of persistent lava lake activity for nearly 10 years (e.g., Patrick et al. 2020). In 2018, magma moved from the summit at Halema‘uma‘u to the Lower East Rift Zone, causing an explosive collapse of the crater, increasing caldera size (Neal et al. 2019). Following the collapse, a water lake formed in the bottom of the caldera from July 2019 to December 2020. This feature was short-lived, as magmatic activity returned to the summit on December 20, 2020 (USGS 2023b).

Kīlauea has had eight eruptions since 2018, six of which have occurred within the summit caldera (Mulliken et al. 2024) (Fig. 2). These recent summit eruptions range in longevity from a few hours to >1 year. Eruptions that persist for >1 week have showcased dynamic perched levee systems that breach and/or fail and then restabilize as the lake level oscillates. Eruptions that extend beyond a month show endogenous growth with expansion of the shallow magma reservoir extending beyond the footprint of the active lake surface (Patrick et al. 2022; Younger et al. 2024). The current eruption in Halema‘uma‘u crater began in December 2024 with 23 episodes as of the end of May 2025. These eruptive episodes are characterized by spectacular lava fountains up to 300 m in height and accompanying lava flows.

The focus of this study is on data collected during the January 5–March 7, 2023, eruption. This 61-day-long eruption maintained relatively stable lava lake activity until February 19, 2023, and then transitioned into a lower-energy phase ending on March 7, 2023. Our GPRI measurements were taken on January 19, 2023, and captured oscillations in the lake level, several breaches in the levee system, and oozing of lava out of the passive lake margins. Figure 2 shows the lava lake surfaces from 2018 through 2023.

## Terrestrial radar interferometry

Satellite microwave techniques have revolutionized volcano monitoring, with GNSS (Global Navigation Satellite Systems) providing continuous and accurate surface deformation data, albeit with limited spatial resolution, and satellite InSAR (Interferometric Synthetic Aperture Radar) providing exquisite spatial resolution, with a temporal sampling of several days or better, depending on satellite constellation. Terrestrial Radar Interferometry (TRI) fills an observational gap, providing precise (mm-scale) surface displacement data with high (meter-scale) spatial resolution over broad (kilometer scale) areas, and with temporal resolution (minutes) that far exceeds any satellite constellation currently flying (Di Traglia et al. 2014b, 2021; Schaefer et al. 2019). Radar backscatter data from TRI has been effectively used for volcano monitoring, offering insights into surface deformation, changes in magmastatic pressure, and precursors to crater-wall collapse (Di Traglia et al. 2014a; Calvari et al. 2016).

**Fig. 3:**
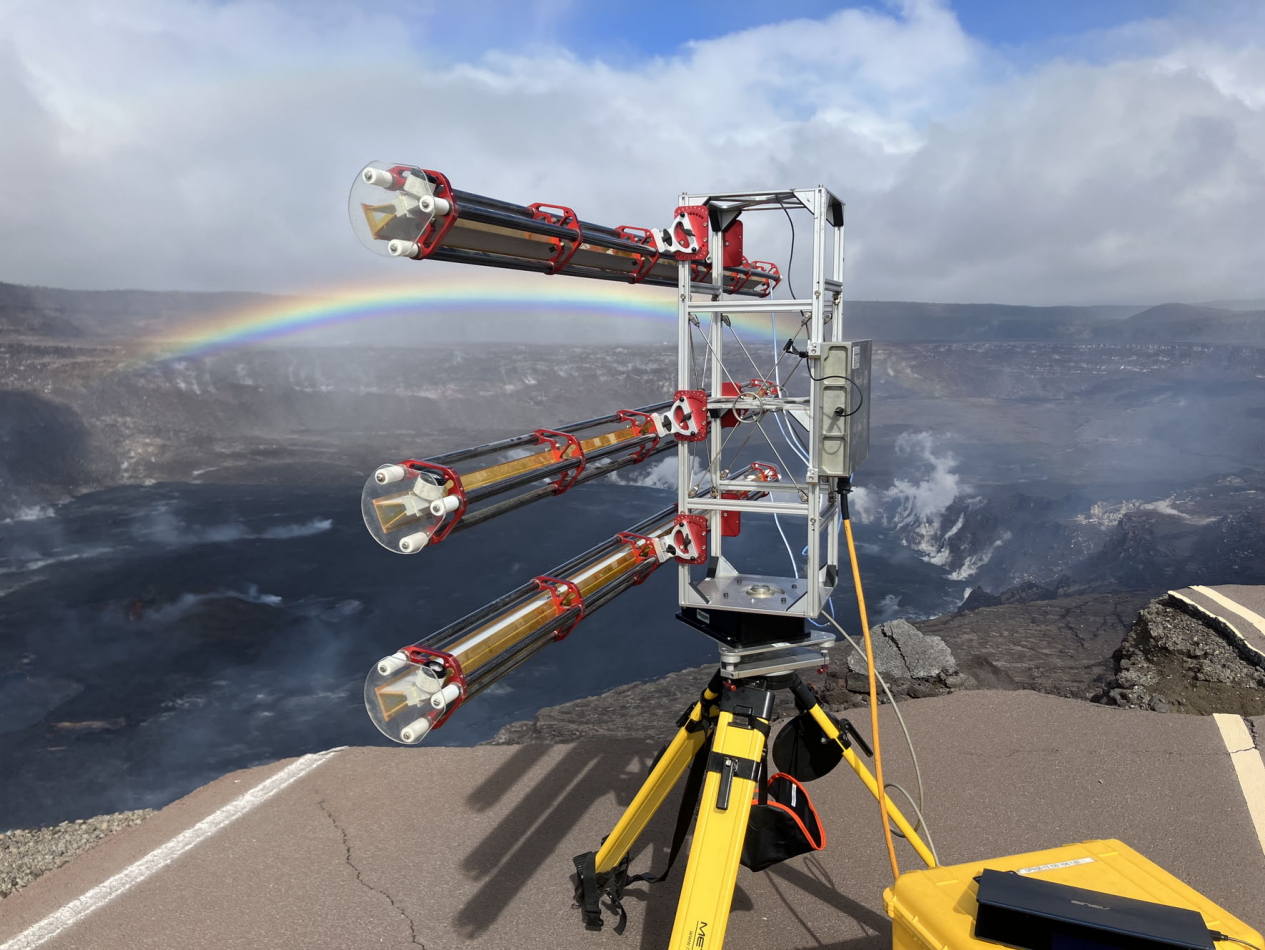
GPRI on tripod rotates and scans Halema‘uma‘u lava lake. The upper antenna transmits the radar signal, while the middle and bottom antennas receive the backscattered signal

Our TRI is a Gamma Portable Radar Interferometer (GPRI), a scanning real aperture radar operating at Ku-band (17.4 mm wavelength), sensitive to LOS displacement at the level of $\sim$1 mm or better (e.g., Fig. 3; Werner et al. 2008). On January 19, 2023, we set up this instrument on a tripod $\sim$1 km from the main deformation area in the southwest portion of Kīlauea caldera (19.39974 N, 155.28608 W; Fig. 1), scanning the Halema‘uma‘u lava lake every 90 s. The GPRI scanned across a 95° arc with a center azimuth angle of 21° from North. Raw radar echoes were converted to single look complex (SLC) images, including phase and amplitude information. We collected a total of 60 scans over a 1.5-h observation period (9:30 AM to 11:10 AM Hawaii Standard Time, hereafter HST).

The slant range resolution of the GPRI is defined by its 200 MHz bandwidth, equal to $\sim$75 cm and independent of range. The azimuth resolution depends on the slant range and antenna beamwidth, $\sim$7 m at 1 km slant range (Werner et al. 2008). The GPRI generally operates with one transmitting antenna (top in our setup, Fig. 3) and two receiving antennas (middle and bottom, Fig. 3). This allows topographic mapping by considering phase interferometry between the two-receiver baseline (e.g., Xie et al. 2016; Deng et al. 2019; Sadeghi Chorsi et al. 2022) or active deformation mapping by considering phase interferometry between successive image scans, using the transmitting antenna and one of the receiving antennas. Given the short (90 s) period between scans, atmospheric variation is minimal, and it is possible to construct precise deformation time series with extremely high temporal resolution. This approach also avoids contamination by phase differences due to topography (e.g., Voytenko et al. 2017). The images are co-registered to the first data (09:34:00 AM HST scan), hence there is no need for a precise external DEM. The images are geocoded based on the GPRI’s setup location, azimuth angle, and spatial resolution. This technique eliminates the need for an external DEM in the data processing. The area covered by our GPRI scans extends up to 8 km from its setup location, although radar decorrelation occurs beyond a few km due to vegetation cover and atmospheric effects. For this report, we focus on data acquired up to 2 km from the radar, ensuring reasonable spatial resolution and phase coherence over the deformation area.

## Radar data processing

**Fig. 4:**
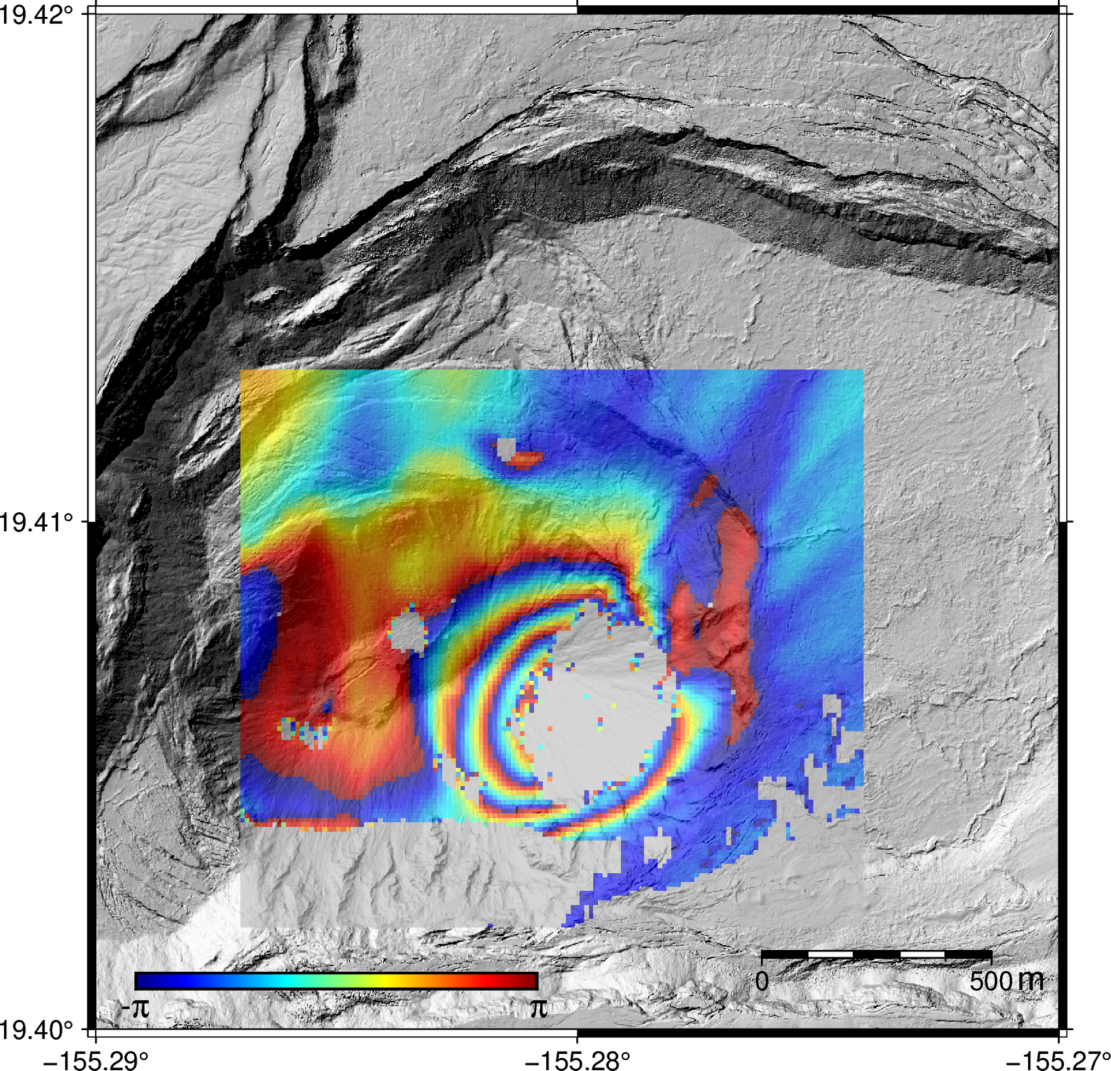
Wrapped interferogram showing phase change between 9:34 HST and 11:06:30 HST. By convention, each color cycle (“fringe”) represents a change in the line of sight (LOS) between the radar and a given pixel of one-half the radar wavelength, in this case 8.7 mm

We generate interferograms from single look complex (SLC) images using the GAMMA software package (Werner et al. 2000). The reference image is the first image acquired (SLC collected at 09:34:00 AM HST). Each of the remaining images is considered the secondary image in the interferometric pair, defining a deformation time series. We spatially averaged (multi-looked) the GPRI data using a 10$\times$1 (Range $\times$ Azimuth) window to reduce noise. We then used an adaptive filter (exponential parameter 0.6, window size 32) to reduce phase noise (Goldstein and Werner 1998). Figure 4 shows an example interferogram.

Phase unwrapping for individual filtered interferograms was performed using a minimum-cost-flow algorithm (Chen and Zebker 2002). Areas with a correlation lower than 0.4 were masked during the phase unwrapping step. The reference point with correlation > 0.8 (from beginning to end of data collection) was selected well outside the deformation area. Unwrapped interferograms were georeferenced to 10 m pixel spacing by considering the setup location and azimuth angle of the GPRI. The unwrapped phase was then converted to LOS displacement through Eq. 1 and projected to a UTM coordinate system for further processing.

1 $$\begin{aligned} LOS = \Delta \phi \times (\frac{-\lambda }{4\pi }) \end{aligned}$$

where $\Delta \phi$ is the unwrapped interferometric phase and $\lambda$ = 1.74 cm is the GPRI wavelength.

We used the Copernicus GLO-30 DEM (Sinergise 2021) and changed its projection and pixel size to match our interferograms, calculating the local radar incidence angle for each pixel in the deformation area. Local incidence angle is defined as the angle between the nadir direction and the LOS vector. This angle was computed by calculating the height difference and horizontal distance between the GPRI’s antenna and a given pixel. The local incidence angle ($\theta$) is defined as:

2 $$\begin{aligned} \theta = \arctan (\frac{d}{\Delta H}) \end{aligned}$$

where *d* is the horizontal distance and $\Delta H$ is the height difference between GPRI receiver and the corresponding pixel in UTM-WGS-84 coordinate system. The local incidence angle for our deformation area is between $\sim$75° to $\sim$89°, reflecting the data acquisition geometry of the GPRI.

The Copernicus GLO-30 DEM was only used to estimate the incidence angle in the lava lake area, where the terrain is relatively flat and can reasonably be approximated as a planar surface. This valuable dataset was not used for coregistration or georeferencing the radar images. The GPRI system is capable of generating a DEM—as a supplementary product in some cases—for each acquisition thanks to its dual-antenna configuration. In radar interferometry-based deformation studies, zero-baseline observations are preferred to avoid the effect of DEM on the radar phase. Our GPRI allows us to obtain such zero-baseline observations over a short time interval, effectively eliminating DEM-related errors and reducing tropospheric effects.

The GPRI transmits and receives radar waves while rotating. The heading angle (clockwise angle from North, defining azimuth) for each image line differs and is related to the GPRI’s setup azimuth angle, total arc angle, and number of azimuth samples. In our case, we collected data from –45° to 50° from the GPRI’s setup azimuth angle, with 940 azimuth samples. It is important to note that the GPRI’s long antennas (2 m) can be susceptible to instability in windy conditions, potentially affecting data quality. To mitigate this, we mounted the GPRI on a short, wide-legged tripod fixed to the ground throughout data collection to minimize vibrations. Moreover, we excluded a few SLCs that showed signs of radar phase decorrelation and phase ramps. We also limited the interferometric analysis to coherent pixels, which helped reduce the impact of any residual vibrations on the results.

## Results

**Fig. 5:**
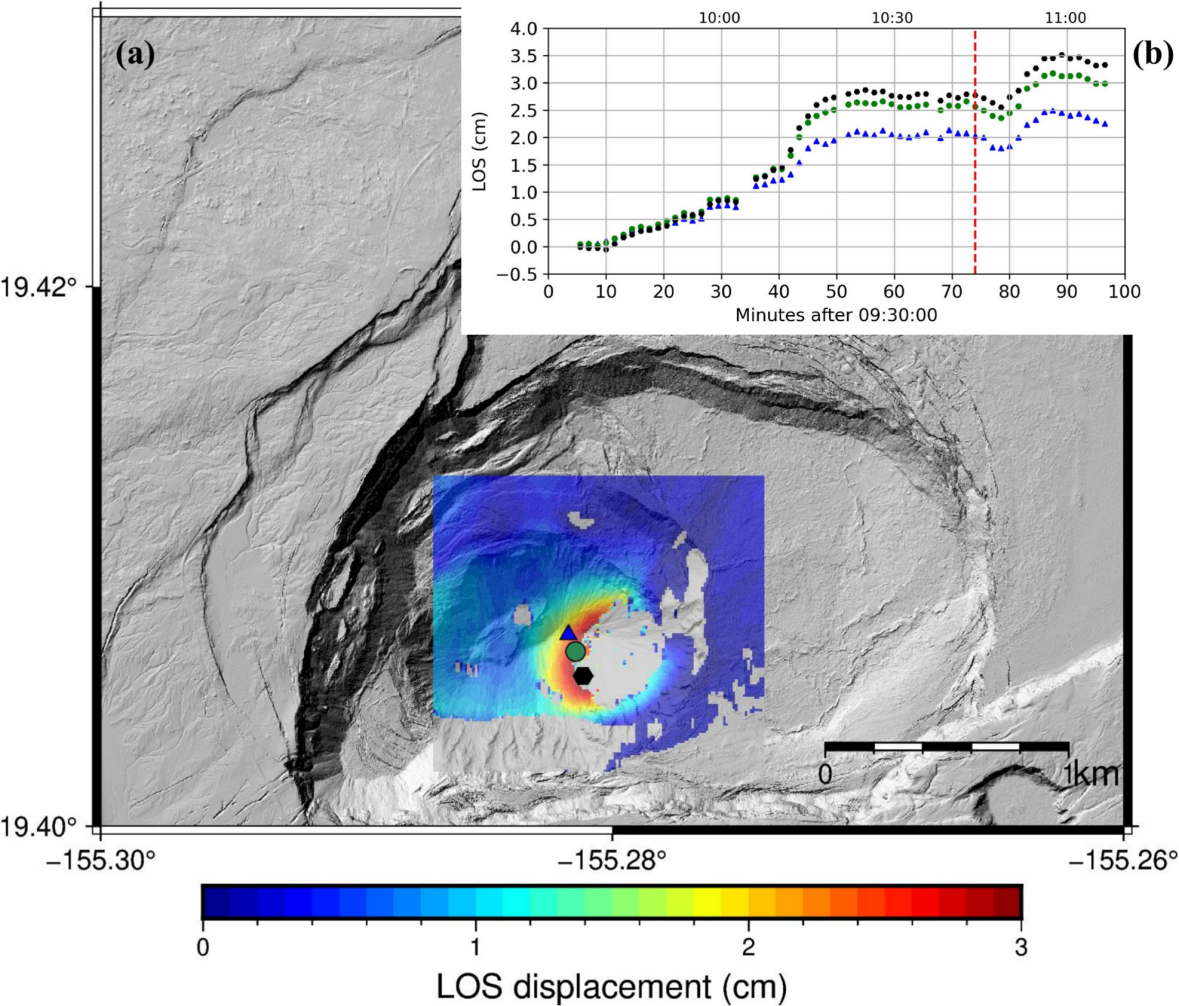
(**a**) LOS displacement map from 09:34:00 to 11:06:30 AM HST on January 19, 2023. Positive values mean displacement towards GPRI. Markers represent coherent displaced areas whose cumulative LOS displacements are shown on the insert (Fig. 5b). (**b**) LOS displacement referenced to 09:34:00 AM HST. The red dashed line indicates the time of failure of a levee on the perched pond (10:44:00 AM HST, see Fig. 6). A video showing a time series of these data as well as individual interferograms can be accessed at (http://labs.cas.usf.edu/geodesy/, or https://www.youtube.com/@USFGeodesyandRemoteSensingLab)

Our GPRI data captured a deformation event on the surface of the lava lake starting just a few minutes after the beginning of data collection and lasting about 90 min. Figure 5 shows cumulative LOS displacement for the study area (5a) and displacement time series for a few pixels at or near the area of maximum deformation (5b). Using these time series, we divided the deformation into seven stages based on deformation type (inflation, deflation, and stability) and rate (Table 1). These stages are used in our subsequent models (Section “Deformation source model”).

The surface of the lava lake inflates at an increasing rate for the first $\sim$45 min (Stages 1–3), then remains stable for $\sim$25 min with no significant displacement (Stage 4). Next, a small amount of subsidence occurs over 3 min (Stage 5). Inflation resumed at 10:47 for about 9 min (Stage 6) before leveling off at 10:56 to the end of observation time (Stage 7). The lava lake experienced a total of 3.5 cm of LOS displacement in about 1.5 h, with deformation rates ranging from –4 to 7.3 cm/h in the LOS direction for the individual stages. Figure 5b demonstrates that the deformation rate remained relatively uniform across all observed points for approximately 35 min after the initiation of data collection; subsequently, a noticeable change in deformation rates was observed, indicating the onset of spatial and temporal variability in the deformation behavior.

This is a small, local deformation event—other nearby geodetic data (GNSS, tilt) do not show obvious perturbations at this time (e.g., the tiltmeter closest to our deformation area; Fig. S1). Similarly, available seismic data do not show anomalous activity consistent with the small magnitude and shallow nature of the deformation (Section “Deformation source model”), as the tremor signal remained constant in the hours before, during, and after radar data collection as confirmed by USGS-HVO personnel (N. Bennington, Personal Communication, May 7, 2025), with only minor volcano-tectonic events scattered throughout the period and a single long-period event recorded at 10:23 HST.

### Subsidence event

**Fig. 6:**
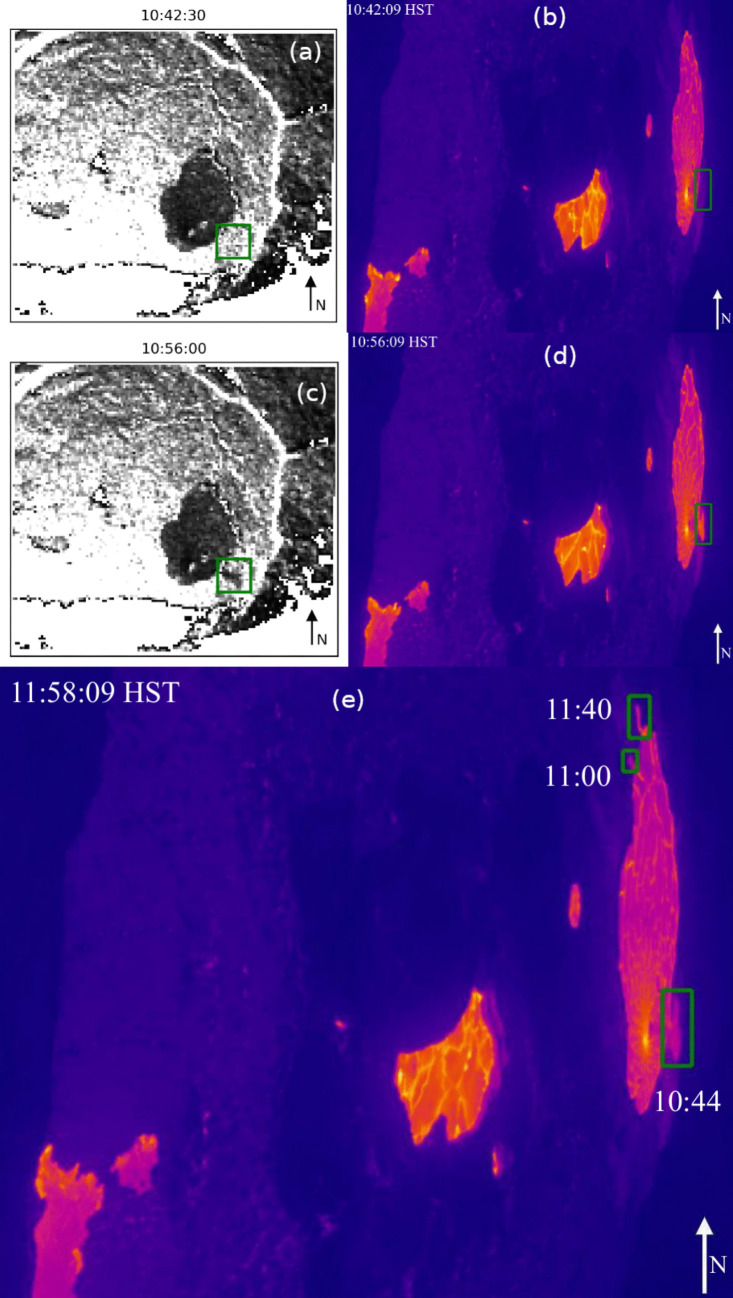
Radar backscatter images (**a**, **c**) and thermal images (**b**, **d**) for time steps before and after a breach in the lava lake surface at 10:44:00 HST and subsequent lava flow (green box). The thermal image in panel (**e**) was captured $\sim$50 min after GPRI data collection and shows three breaches (green boxes). GPRI was set up southwest of the main deformation area, while the thermal camera is located west-northwest of this area. Thermal images are rotated 90 degrees clockwise to be similar to radar images

After the initial period of inflation, the lava lake surface experienced a subsidence event (Stage 5, above) from 10:44 to 10:47 HST (Fig. 5). The beginning of this subsidence event coincided with an overflow and eventual levee breach (Fig. 6). The breach and subsequent eruption of lava likely caused a loss of pressure and volume in the magma source and corresponding subsidence.

Inspection of the thermal imagery outside the time window of our data suggests that these small lava breakouts are common (Fig. 6e) and likely limit pressure build-up in the system as observed by one of us (EG) and captured by both thermal imagery and radar backscatter images (Fig. 6).

Radar backscatter depends on imaging angle, surface roughness, and dielectric properties. The GPRI imaged the lava lake surface from the side, and the new lava surface is relatively smooth, so most of the radar energy is reflected away from the receiver. Hence, new lava appears dark in our Ku-band imagery. The larger lava surface and the new breach appear elongated in the thermal imagery compared to the radar backscatter imagery. The thermal imagery is acquired by a camera (F1cam, Fig. 1) on the west side of the caldera and, like the radar, has highly oblique imaging geometry. The radar imagery has been geometrically corrected for this effect. The thermal imagery has not and thus appears distorted.

The GPRI’s shallow incidence angle means that our scalar LOS measurements are more sensitive to horizontal motion compared to vertical motion. However, it is likely that the deformation event we imaged induced displacements in all three spatial dimensions. This limitation is addressed in the next section.

## Deformation source model

### Rheology

Estimated source depths in most dislocation models can be sensitive to the distribution of material properties in the medium (e.g., Manconi et al. 2007; Masterlark et al. 2016), although high-accuracy three-dimensional vector geodetic data provide strong constraints (e.g., Dixon et al. 1997). We expect the material properties of the lava lake to vary significantly near the surface, where thermal gradients are high. The top of the lava lake likely comprises a thin, rigid, or semi-rigid horizontal crustal cap of overlapping lava flows overlying a convecting system of molten to partially crystallized gas-rich magma whose properties vary in three dimensions and time. The thickness of the crustal cap reflects the time since the last major eruption, with the cap cooling mainly by conduction. The deformation event we observed in 2023 likely reflects the upward motion of gas-rich magma beneath this rigid or semi-rigid cap, with the magma eventually reaching the level of neutral buoyancy (e.g., Crozier and Karlstrom 2022).

The ideal model for this deformation event would account for strong rheological gradients in the convecting system, including its time-varying nature. Given the lack of information on these rheological properties, our scalar LOS data, and the lack of complimentary geodetic or seismic data to define source shape or depth, we therefore applied the simplest possible model, namely dislocations in an elastic half-space, as has been done previously for Kīlauea (e.g., Anderson and Poland 2016). Since the period of maximum deformation is short ($\sim$1 h) the elastic approximation is probably valid. Future models could address the influence of complex rheology (e.g., Newman et al. 2001, 2006). Limited independent information provides some constraints on source depth, as described in the next section.

### Modeling approach

Data were inverted assuming various dislocation source geometries in a uniform elastic half-space (Okada 1985). We tested simple Mogi (point source) models (Mogi 1958), as well as a prolate spheroid source (Yang et al. 1988) and a variety of simple dike and sill sources. In all cases, planar dislocations with horizontal or near-horizontal geometries (sill-like sources) are preferred. To reduce the computational burden, the LOS displacement data from each selected interferogram were down-sampled. The down-sampled data for each pixel, along with its corresponding heading and incidence angles (see Section “Radar data processing”) were then inverted to estimate a likely set of source parameters and their associated uncertainties (Di Traglia et al. 2015).

Our final suite of models assumes a horizontal dislocation geometry with spatially variable inflation, highly smoothed to reflect the non-uniqueness of our scalar data. We tested a range of source depths via forward models based on a simple cooling model and geometric considerations. For a minimum depth estimate, we assume that sill depth must be at least of the order of the crustal thickness, i.e., the sill ponds at the base of the rigid or semi-rigid crust of the lava lake. We make a first-order approximation of crustal thickness using the conductive cooling model for Kīlauea Iki (Hardee 1980), assuming an essentially stable lava lake surface since 2021. At Kīlauea Iki, downhole temperature measurements extending to the melt indicated that for the first several years the crust of the lake solidifies according to the classical square root of time law (Peck et al. 1964; Wright et al. 1976; Macdonald 1972). After six years, the crust began to solidify at a near-constant rate. For the initial period, from Hardee (1980):

3 $$\begin{aligned} D = 1.21\times 10^{-3}\sqrt{\tau } \end{aligned}$$

where *D* is the distance from the surface to the solidification front in meters and $\tau$ is the time in seconds. For Halema‘uma‘u, assuming a relatively constant lava lake surface from September 2021 to January 2023 gives a $\tau$ of 17 months ($4.4\times 10^{7}$ sec), yielding a crustal thickness of order $\sim$10 m. Continued magmatic intrusions and eruptions would add both heat and mass to the system and could reduce this estimate further.

An alternate approach recognizes the possibility of a “layer cake” rheological structure from the several eruptions into the lava lake since 2018 (Fig. 2). The deformation event we record may reflect gas-rich magma ponding near a relict viscosity contrast, such as an earlier lava lake surface, implying source depths of order 100 m. We therefore tested source depths between 10 and 100 m.

#### Sill deformation inversion

Surface displacement due to multi-patched sill deformation can be written as Eq. 4:

4 $$\begin{aligned} d_{i} = \sum _{i,j}^{} u_{tn}^{j} g_{tn}^{i,j} \end{aligned}$$

where $d_{i}$ is the $i^{th}$ down-sampled LOS displacement on the surface, $u_{tn}^{j}$ represents tensile motion of the $j^{th}$ patch of the sill segment, and $g_{tn}^{i,j}$ is the data kernel that corresponds to the geometry and physics of the problem. To add prior information into the solution, we apply a smoothness constraint by minimizing the two-dimensional second-order derivative for each sill patch (Jónsson et al. 2002; Menke 2018; Deng et al. 2020; Sadeghi Chorsi et al. 2022b, a).

These equations can be written in matrix form:

5 $$\begin{aligned} d = Gm \end{aligned}$$

where *d* includes down-sampled LOS displacement vector and a priori information, *G* contains Green’s function based on Okada (1985) model and second-order derivative with smoothness coefficients, and *m* is the model parameter vector which represents the tensile motion of each sill patches. *m* can be estimated using least square:

6 $$\begin{aligned} m = \left[ G^{T}G \right] ^{-1}G^{T}d \end{aligned}$$

We divide the 800 m $\times$ 800 m sill into 20 $\times$ 20 patches (each patch size is 40 m) and estimate the opening for each patch considering different depths.

**Fig. 7:**
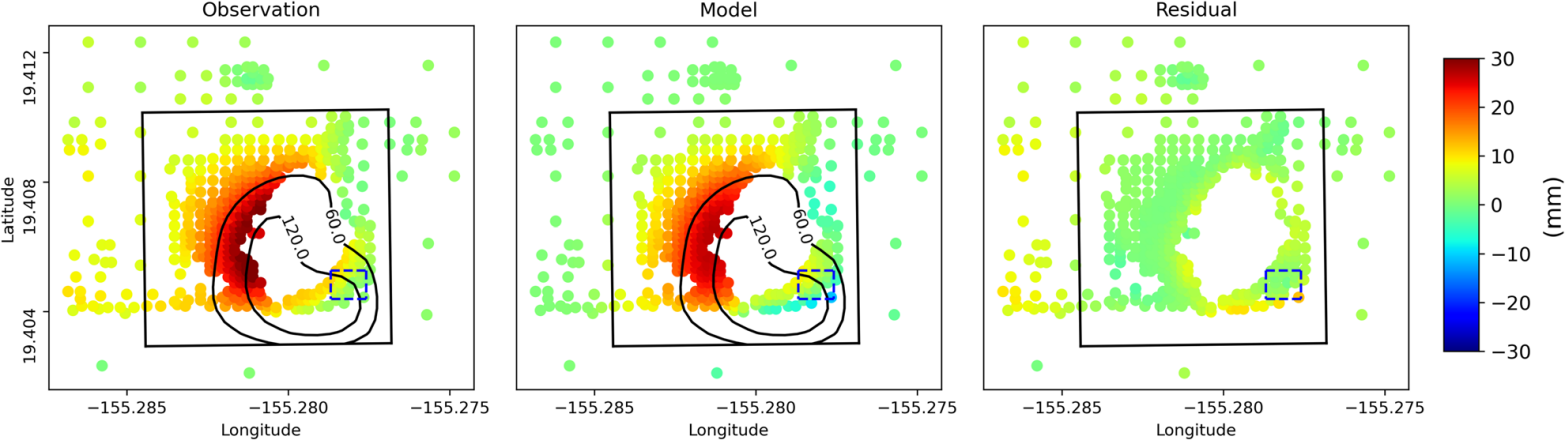
LOS displacement (**left**), model (**middle**), and residual (**right**) for sill opening model source for interferogram 09:34:00 to 11:06:30. Each point represents the down-sampled LOS. White areas indicate decorrelation. Positive values indicate LOS displacement towards the GPRI. Black box represents sill geometry projected to the surface. Dashed box shows breach area (Fig. 6). Contour lines outline the sill deformation area

**Fig. 8:**
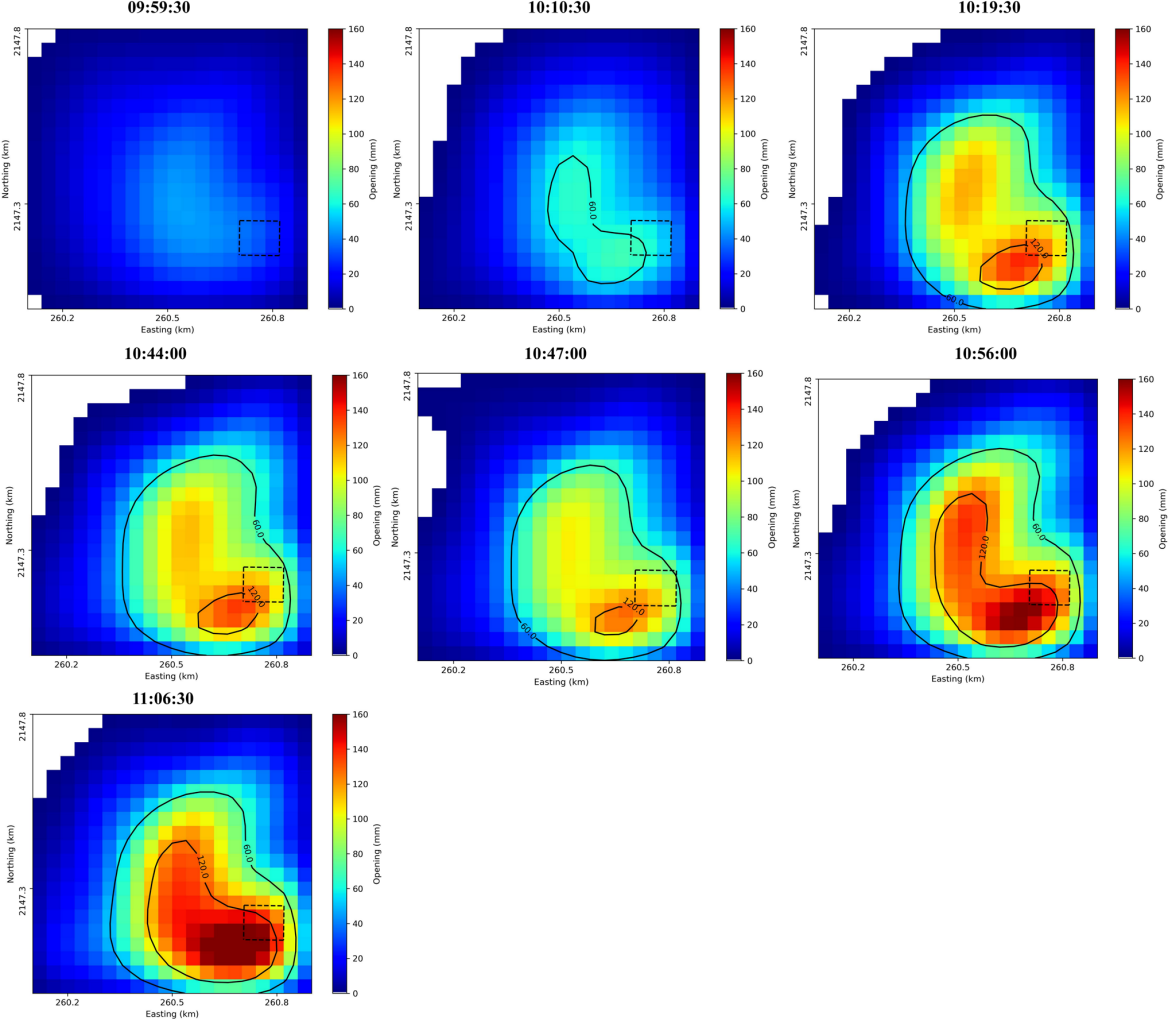
Sill opening model for seven stages (Table 1) with respect to first data (09:34:00). Dashed box shows the breach area (Fig. 6). Contour lines outline the highly deformed area. Plots are shown in UTM-WGS84 coordinates for improved spatial computation

### Modeling results

Figure 7 shows observation, model, and residual LOS for the sill opening model for the entire period 09:34:00 to 11:06:30, assuming sill depth = 50 m. To investigate the deformation stages, we adopt an approach based on a fixed source geometry while allowing the opening of the sill to vary over time to best match the LOS data at different stages. This method, which assumes a constant geometry with time-varying deformation intensity, has been previously applied in similar geodetic studies (Giudicepietro et al. 2024; Di Traglia et al. 2023). This allows us to estimate volume changes for the different stages of the deformation event, assuming piece-wise linear behavior over short intervals of time (Fig. 8). Table 1 lists the series of interferograms used for these stage calculations.

Figure S2 shows corresponding results for the other assumed depths. Minimum and maximum root mean square error (RMSE) between the LOS data and models for different stages ranges between 1 mm (for 09:59:30) and 4 mm (for 10:56:00). Note that regardless of depth, the sill closely matches the displaced surface, reflecting its shallow assumed depth.

Deformation starts near the center of the sill a few minutes after data collection begins, then migrates to the southeast where the breach occurred at 10:44:00 (dashed box). The maximum opening occurs within 100 m of the breach, reaching a value of 179 mm at 11:06:30.

### Volume calculation

**Fig. 9:**
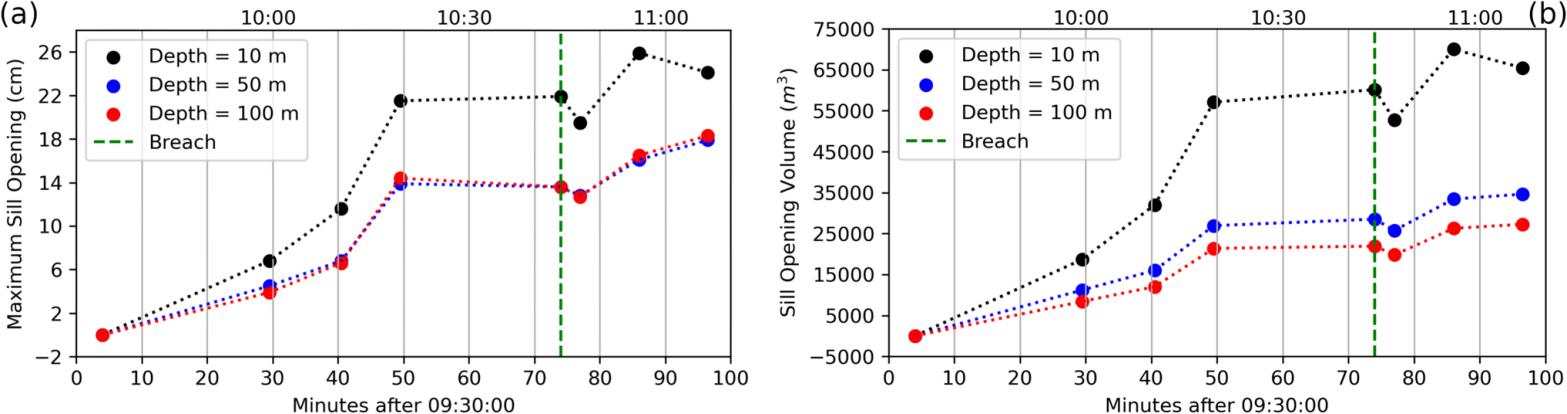
(**a**) Maximum sill opening values from inversion of the selected interferograms representing different deformation stages and (**b**) volume change during opening variation of the sill. Green dashed line shows time of breach

Sill opening volume is calculated by multiplying the length, width, and opening value of each patch and then summing the results (Table 1, Fig. 9). The sill volume change for each stage ranges from $\sim$–2,760 to $\sim$11,216 $m^{3}$. The overall volume change is 34,624 $m^{3}$ for the total 90 min event. The deflation event was relatively small, $\sim$8% of the inflation volume. Note that these volume amounts depend on the deformation model (Fig. 9). The volume flux during inflation ranges from 7.2 to 20.2 $m^{3}/s$, representing addition of gas-rich magma, i.e., not dense rock equivalent.

## Discussion

Our best-fit model for the observed deformation event calls for a small volume recharge of gas-rich magma into a shallow sill in the lava lake near the surface of the lava lake, in between the 2020 and 2023 eruption fill depths (Fig. 2). The volume change rates in Table 1 are similar in an order-of-magnitude sense to the long-term volume change rates tabulated by Wadge (1982) for a variety of steady-state volcanic systems, including Kīlauea. This suggests the possibility that the event we imaged occurs frequently, but is missed given current observational limitations.

We can compare the volume of the observed intrusion to the total lava lake volume. Using the USGS profile as a guide, we can approximate the total lava lake volume as a cone ($V_{cone} = \pi r^{2}h/3$) with a radius of 500 m and height of 350 m, yielding a total volume of $\sim$9.2$\times$ $10^{7}$ $m^{3}$ (Mulliken et al. 2024). The total volume change over the 90 min observation period was 34,624 $m^{3}$, about 0.038% of the total lava lake volume. Perhaps this represents the minimum volume of gas-rich magma capable of overcoming viscous forces to ascend through the lava lake. These intrusions are small, but perhaps frequent enough to help maintain the lava lake in a near-steady-state.

**Table 1:** Surface deformation and volume flux data for each stage

Stage	Time interval	Minutes after 9:30 (duration)	Surface deformation	Surface displacement (*cm*)	Rate (*cm*/*h*)	Volume change ($m^{3}$)	Volume change rate ($m^{3}/s$)
1	9:34:00–9:59:30	4–29.5 (25.5)	Inflation	0.8	1.9	11,215.87	7.33
2	9:59:30–10:10:30	29.5–40.5 (11)	Inflation	0.6	3.3	4,785.58	7.25
3	10:10:30–10:19:30	40.5–49.5 (9)	Inflation	1.1	7.3	10,934.59	20.25
4	10:19:30–10:44:00	49.5–74 (24.5)	Stable	0.1	0.2	1,580.53	1.08
5	10:44:00–10:47:00	74–77 (3)	Subsidence	$-$0.2	$-$4.0	$-$2,763.08	$-$15.35
6	10:47:00–10:56:00	77–86 (9)	Inflation	0.8	5.3	7,678.41	14.22
7	10:56:00–11:06:30	86–96.5 (10.5)	Stable	$-$0.2	$-$1.1	1,192.48	1.89

In order to estimate the amount of magma supplied to the lava lake and to compare to longer time interval magma supply rates for Kīlauea, we need to correct for the gas content to derive dense rock equivalent (DRE). The gas-rich nature of magma within Halema‘uma‘u is well documented. For example, very-long-period (VLP) seismicity and continuous gravity data indicate that magma in the lava lake is highly porous with an average porosity of $\sim$70% (Crozier and Karlstrom 2022; Poland and Carbone 2016, 2018). The uppermost parts of the lava lake have even higher porosities (up to 92–96%) and lower densities (100–200 $kg/m^3$) forming a ‘magmatic foam’ cap consistent with the ejection of reticulite clasts ($\sim$310 $kg/m^3$) from the vent during explosive eruptions (Carey et al. 2013, 2015; Mangan and Cashman 1996). If we assume the gas content of the intruding magma is 70%±20%, then the modeled volume change should be decreased by factors of 2 to 10 to compare to magmatic DRE values.

The DRE of the total intruded gas-rich magma with 70%±20% gas would be about 6,800 $m^3$ (2,500 to 11,000 $m^3$) over the 90-min observation period. Estimates for the daily magma supply rates for Kīlauea are of order 217,000±10,000 $m^3/day$ (Denlinger 1997) to 388,800± 129,600 $m^3/day$ (Anderson and Poland 2016). Thus, the observed intrusion event represents a tiny fraction (< 5%) of the daily magma supply for the Kīlauea system. We suspect these are frequent events, unnoticed until now because of observational challenges. More frequent observations will be required to assess their numerical importance.

The addition of both heat and mass is required to maintain a lava lake in a steady-state condition, all while adding little or no long-term volume. The intrusion of deeper-sourced magma provides an infusion of heat that compensates for the loss of heat via conductive and advective cooling from the top. The addition of gas-rich magma can compensate for the loss of accumulated gas from the lava lake top and the loss of magma to deeper levels when dense degassed magma drains in back into reservoirs below (e.g., Dixon et al. 1991; Suckale et al. 2018; Wallace and Anderson Jr 1998). Outgassing of the lava is accomplished through continuous passive gas loss, spontaneous high-energy bursts, and triggered lower-energy bursts. Relatively high-energy outgassing of this foam layer is most often accomplished by spontaneous gas pistoning events (e.g., Patrick et al. 2019; Vergniolle 1996; Vergniolle and Jaupart 1986, 1990; Carey et al. 2015). Gas pistoning refers to a cyclic rise and fall of the lava lake surface associated with fluctuations in outgassing and spattering. Previous literature describes these cycles as lasting minutes to several hours with surface height changes of meters to several 10 s of meters. Our new data document that smaller events, with surface displacement, changes as small as a few centimeters, also occur. They could be quite common but to our knowledge have not been previously observed.

In summary, we interpret the respective stages of the observed deformation as follows. Inflation during Stages 1, 2, and 3 results from the intrusion of gas-rich magma into a sill just below the lava lake surface with the inflation rate increasing from 1.9 to 7.3 cm/h over the 90-min observation interval. An observed lava breach at the beginning of stage 5 coincides with a change from inflation to deflation. The deflation is modeled as a loss of $\sim$10% of the previously intruded volume (Stages 1 to 4) from the sill. Deformation data alone, however, cannot distinguish a loss of gas from the sill versus a loss of accumulated foam at the top of the lava lake. As the sudden gas release tapers off, inflation resumes as the dominant signal.

Data collection with the GPRI system presents inherent challenges, particularly due to the requirement to be positioned on elevated terrain. These constraints can limit the spatial coverage, as radar shadowing may obscure certain areas. As a result, we cannot conclusively determine whether other sections of the lava lake—such as the westernmost segment—were involved in the deformation event. Moreover, it is possible that the observed 90-min deformation was part of a longer deformation cycle.

## Conclusions

 

1. Ground-based radar interferometry has been used for the first time to detect a small-scale intrusive event into Kīlauea’s lava lake. This technique allows observation of phenomena at a scale and resolution that has not been previously possible.
2. We model the intrusion as a shallow sill at depths between 10 m and 100 m, with dimensions of order 500 m by 500 m.
3. We suggest that such small volume intrusions of gas-rich magma are likely frequent and help to provide the necessary flux of heat and mass to compensate for cooling, outgassing, and recycling of dense degassed magma to sustain steady-state summit lava lake activity.

## Supplementary information

The article includes supplementary files.

## Supplementary Information

Below is the link to the electronic supplementary material.

Supplementary file 1 (pdf 11433 KB)

## Data Availability

The Copernicus GLO-30 digital elevation model is publicly available through (Sinergise 2021). Interferograms and multi-looked images were formed using the GAMMA software packages (Werner et al. 2000). Figures 4 and 5 are made using PyGMT (Tian et al. 2025). Radar images and its products are available upon request.

## Code availability

Python data processing codes are available upon request. The Okada wrapper for Python used in the inversion can be accessed at https://github.com/tbenthompson/okada_wrapper.git.
