# Supplementary material for: Small intrusions may help maintain Kīlauea’s lava lake
Source: Bull Volcanol. 2025 Jul 7;87(8):62. doi: 10.1007/s00445-025-01847-8 (PMC12234635; doi:10.1007/s00445-025-01847-8)
Supplement: Supplementary file 1 — (pdf 11433 KB) [file 445_2025_1847_MOESM1_ESM.pdf]

The material in this supplement shows supporting data for the paper Small Intrusions Help Maintain Kīlauea's Lava Lake

Introduction

Figure S1 compares the radar displacement data to tilt data captured around the same time and several hours before and after the radar data, recorded at a tiltmeter site (Station UWE, data courtesy USGS). This site is located 2.4 km from the TRI setup location, and 1.9 km from the area with the maximum LOS deformation. The TRI is 0.8 km away from the area with the maximum LOS deformation. There is no obvious correlation between the two time series, suggesting that surface deformation of the lava lake reflects a local, shallow source.

Figure S2 compares the radar observations of surface deformation to several models consisting of an inflating sill at various depths, and corresponding residuals (observation minus model). All three models show similar (and low) residuals, indicating that the scalar radar data cannot uniquely constrain the depth of deformation. Note that the 50 m and 100 m depth model have a well-defined center of maximum deformation that is close to the surface location of the lava break out (small dashed box).

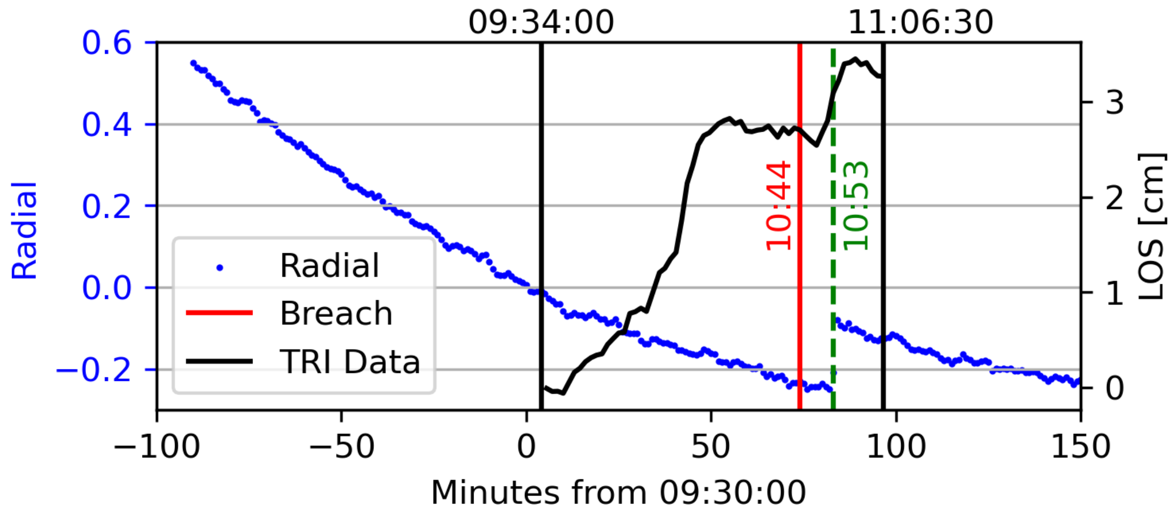

Figure S1. Radial component of tiltmeter located northwest of deformation area shown in blue (Site name: UWE; Lat: ~19.421 N, Lon: ~155.291 W). Black line shows the LOS time series for one pixel with large displacement (See Figure 5). Red line shows when breach happens.

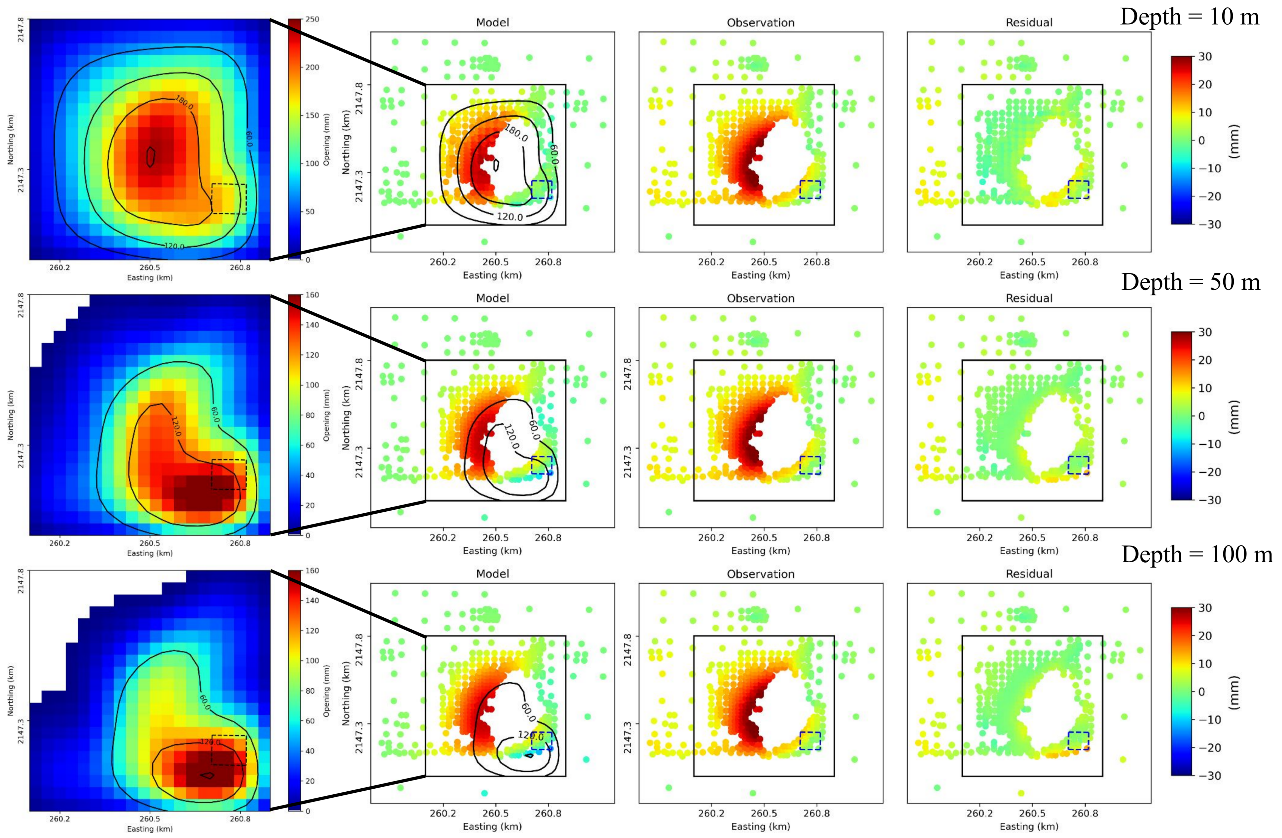

Figure S2. Zoomed-in model (left panels), model, observation, and residual for inverted sill model for different depth (Top: depth = 10 m, middle: depth = 50 m, and bottom: depth = 100 m).
